# Perspectives in immunotherapy: meeting report from the “Immunotherapy Bridge”, Napoli, November 30th 2016

**DOI:** 10.1186/s12967-017-1309-2

**Published:** 2017-10-11

**Authors:** Paolo A. Ascierto, Bruno Daniele, Hans Hammers, Vera Hirsh, Joseph Kim, Lisa Licitra, Rita Nanda, Sandro Pignata

**Affiliations:** 1 Unit of Melanoma, Cancer Immunotherapy and Innovative Therapy, Istituto Nazionale Tumori “Fondazione G. Pascale”, Via Mariano Semmola, 80131 Naples, Italy; 2 Department of Oncology and Medical Oncology Unit, G. Rummo Hospital, Benevento, Italy; 3 UT Southwestern, Medical Center, Dallas, TX USA; 4 McGill Department of Oncology, McGill University, Montreal, Canada; 5 Medical Oncology, Yale School of Medicine, New Haven, CT USA; 6 Medical Oncology Unit, Fondazione IRCCS Istituto Nazionale Tumori, Milan, Italy; 7 Section of Hematology–Oncology, Department of Medicine, The University of Chicago, Chicago, IL USA; 8 Department of Urology and Gynecology, Istituto Nazionale Tumori “Fondazione G. Pascale”, Naples, Italy

**Keywords:** Immunotherapy, Melanoma, Solid tumors

## Abstract

The complex interactions between the immune system and tumors lead the identification of key molecules that govern these interactions: immunotherapeutics were designed to overcome the mechanisms broken by tumors to evade immune destruction. After the substantial advances in melanoma, immunotherapy currently includes many other type of cancers, but the melanoma lesson is essential to progress in other type of cancers, since immunotherapy is potentially improving clinical outcome in various solid and haematologic malignancies. Monotherapy in pre-treated NSCLC is studied and the use of nivolumab, pembrolizumab and atezolizumab as second-line of advanced NSCLC is demonstrated as well as first line monotherapy and combination therapy in metastatic NSCLC studied. Patients with HNSCC have immunotherapeutic promises as well: the FDA recently approved moAbs targeting immune checkpoint receptors. Nivolumab in combination with ipilumumab showed acceptable safety and encouraging antitumor activity in metastatic renal carcinoma. HCCs have significant amounts of genomic heterogeneity and multiple oncogenic pathways can be activated: the best therapeutic targets identification is ongoing. The treatment of advanced/relapsed EOC remain clearly an unmet need: a better understanding of the relevant immuno-oncologic pathways and their corresponding biomarkers are required. UC is an immunotherapy-responsive disease: after atezolizumab, three other PD-L1/PD-L1 inhibitors (nivolumab, durvalumab, and avelumab) were approved for treatment of platinum-refractory metastatic urothelial carcinoma. Anti-PD-1/PD-L1 monotherapy is associated with a modest response rate in metastatic breast cancer; the addition of chemotherapy is associated with higher response rates. Immunotherapy safety profile is advantageous, although, in contrast to conventional chemotherapy: boosting the immune system leads to a unique constellation of inflammatory toxicities known as immune-related Adverse Events (irAEs) that may warrant the discontinuation of therapy and/or the administration of immunosuppressive agents. Research should explore better combination with less side effects, the right duration of treatments, combination or sequencing treatments with target therapies. At present, treatment decision is based on patient’s characteristics.

## Introduction

Traditional treatment for advanced cancer, like radiotherapy, chemotherapy, or targeted agents, have direct action on tumors to inhibit or destroy them. These modalities, along with surgery, are mostly palliative, with toxicity and only modest improvements in survival in patients with advanced solid tumors. Accordingly, long-term survival rates for most patients with advanced cancer remain low, thus there is a need for cancer treatments with favorable benefit and toxicity profiles that can potentially result in long-term survival.

The immune system plays a critical role in the recognition and eradication of tumor cells (“immune surveillance”), and immunotherapies based on this concept have been used for decades with some success against a few tumor types. However, most immunotherapies were limited by a lack of either substantial efficacy or specificity, resulting in toxicity.

Understanding of the complex interactions between the immune system and tumors leads the identification of key molecules that govern these interactions. This information reported the interest of scientific community in immunotherapy as an evolving treatment modality using immunotherapeutics designed to overcome the mechanisms broken by tumors to evade immune destruction. Immunotherapies have potentially complementary mechanisms of action that may allow them to be combined with other immuno therapeutics, chemotherapy, targeted therapy, or other traditional therapies.

Tumor cells feat multiple complex mechanisms to escape recognition and destruction by the immune system. Tumor cells can actively dysregulate immune cell activity (notably, T cells and natural killer cells, NK cells) through mechanisms including the activation of T cell inhibitory (checkpoint) pathways, such as Cytotoxic T-Lymphocyte Antigen4 (CTLA-4), Programmed Death-1 (PD-1), and Lymphocyte Antigen Gene 3 (LAG-3); inhibition of T-cell activation pathways (e.g., CD137, OX-40, CD40, GITR, HVEM) and/or suppression of NK cell activity. Furthermore, the tumor microenvironment contains various immunosuppressive factors from different sources that may be exploited by tumor cells to escape the immune system.

CTLA-4 is an immunomodulatory molecule that down-regulates T cell-activation. Ipilimumab, a fully human monoclonal antibody that blocks CTLA-4 was the first successfully developed drug of a new class of therapeutics named immune checkpoint inhibitors.

PD1 is another immune checkpoint target expressed on activated T-cells mediating immunosuppression. Its ligands PD-L1 (B7-H8) and PD-L2 (B7-DC) are expressed on many tumour cells, stroma cells and other cell types including leucocytes. The immunosuppressive action of the PD1 receptor is activated in the effector phase of the interaction between T lymphocytes and tumour cells, and the blockade of this receptor seems to be more effective towards T-cell-activation than CTLA-4 blockade.

Anti-CTLA4 agents will act in the priming phase of immune response by inhibiting the interaction between the CTLA4 on T cell and B7 on antigen-presenting cell, while anti-PD1 agents will act on the effector phase by inhibiting mainly the interaction between the PD1 on T cells and PDL1 on tumor cells.

Nivolumab (formerly known as BMS-936558) is a genetically engineered, fully human IgG4 monoclonal antibody with high affinity and specificity for human PD-1. It is engineered to avoid the antibody-dependent cellular cytotoxicity that can lead to T-cell apoptosis and subsequently depletion of activated T-cells. By binding to the PD-1 receptor, it blocks its interaction with PD-L1 and PD-L2 present on the surface of tumor cells and other immune cells notably APC, thereby preventing T-cell inhibition and restoring antitumor immune response.

Pembrolizumab (formerly known as MK-3475) is an engineered humanized IgG4 antibody that also selectively targets PD-1 and has two parts: a variable region sequence of a very high-affinity mouse antihuman PD-1 antibody and a human IgG4 immunoglobulin to avoid antibody-dependent cellular cytotoxicity.

The second Immunotherapy Bridge meeting focused on various cancer types including melanoma, non-small cell lung cancer, renal cell, breast and ovarian carcinoma, and discussed mechanisms of action of single agents and combination strategies and prediction of clinical responses.

## Checkpoint inhibitors in advanced melanoma

After the substantial advances in melanoma, the focus of cancer immunotherapy has expanded to include many other type of cancers, but what we learned from melanoma is essential to progress in other type of cancers, since immunotherapy is potentially improving clinical outcome in various solid and haematologic malignancies.

Presently, targeting immune checkpoints, which normally terminate immune responses after antigen activation, is the focus in the treatment of advanced melanoma.

The long-term survival observed for ipilimumab-treated patients with advanced melanoma was investigated in a pooled analysis of Overall Survival (OS) data from multiple studies: among 1861 patients, median OS was 11.4 months (95% CI 10.7–12.1 months), which included 254 patients with at least 3 years of survival follow-up; median OS was 9.5 months (95% CI 9.0–10.0 months), with a plateau at 21% in the survival curve beginning around year 3; a plateau in the survival curve, beginning at approximately 3 years, was observed and was independent of prior therapy or ipilimumab dose. These data demonstrated the durability of long-term survival in ipilimumab-treated patients with advanced melanoma.

See also Table 1. A first consideration can be drawn after these results. OS is considered the golden standard in oncological clinical trials. However, it is appropriate to ask if this could still be true with therapies which prolong so much survival and it could take years before the phase III trial can be completed to confirm that. Besides, giving the number of new drugs under development for melanoma, competition for scarce number of patients to be enrolled is ongoing [5]. Which can be the best surrogate primary endpoint? Long-term progression free survival (PFS) (1- and 2-year PFS) demonstrated to be a strong surrogate for long-term OS, not confounded by post-progression therapy and predicting long-term benefit [5].

**Table 1:** Summary of the most relevant long-term results in patients with melanoma

Study	mOS (mos)	1-years OS%	2-years OS%	3-years OS%	5-years OS%
CA209-003 [1]	20.3	65	47	41%	35%
CA209-066 [2]	NR	70.7	57.7	NA	NA
Keynote-001 all pts [3]	24.4	66	50	40%	NA
Keynote-001 naïve pts [4]	32.2	73	61	45%	NA

A further lesson learned from melanoma is that immunotherapy targets the immune system not the tumor and therefore offers the potential for activity across multiple tumor types.

Pembrolizumab demonstrates broad antitumor activity in melanoma (N = 655) [4], non-small-cell lung carcinoma (NSCLC) (N = 262) [6], head and neck tumor (N = 132) [7], urothelial (N = 33) [8], gastric (N = 39), triple-negative breast cancer (TNBC) (N = 32) [9], Hodgkin Lymphoma (N = 29) [10], mesothelioma (N = 25) [11], ovarian (N = 26) [12], Small-cell lung carcinoma (N = 20) [13], esophageal (N = 23) [14] carcinoma.

Immunotherapy also offers unique safety profiles, although, in contrast to conventional chemotherapy, boosting the immune system leads to a unique constellation of inflammatory toxicities known as immune-related adverse events (irAEs) that may warrant the discontinuation of therapy and/or the administration of immunosuppressive agents.

The use of these agents is set to increase due to their dramatic impact on survival in a variety of advanced-stage cancers, thus, it is relevant to be well versed with the heterogeneous presentations of irAEs in terms of recognition and management. Early diagnosis and appropriate management are essential to minimize life-threatening complications. Unless an alternate etiology has been identified, it is required to consider all signs and symptoms, and systemic high-dose corticosteroids may be required for severe events, with or without additional immunosuppressive therapy.

The most frequent irAEs regard pulmonary district (pneumonitis by PD1 antibodies), endocrine system (hypopituarims, hyper/hypothiroidism, hypoadrenalism), liver district (hepatitis, transaminitis) and gastrointestinal district (diarrhea, colitis, pancreatitis) and finally the cutaneous district (dermatitis, rash, pruritus, vitiligo). However, real world experience (Italian expanded access programme of ipilimumab) demonstrated the safety profile of ipilimumab was consistent with those found in clinical studies and hospitalization due to adverse event being related to the experience of the sites: more experience less hospitalizations [15].

As far as melanoma treatment is concerned, grade 3–4 adverse events are in the range of 13% (nivolumab) [2], 34% (ipilimumab 10 mg/kg) [16] and 56.5% for nivolumab in combination with ipilimumab [17]. The rate of patients who permanently discontinued for any grade adverse events ranged from 6% (nivolumab) [2] to 31% (ipilimumab 10 mg/kg) or 38.7% in case of combination nivolumab/ipilimumab [17].

**Fig. 1:**
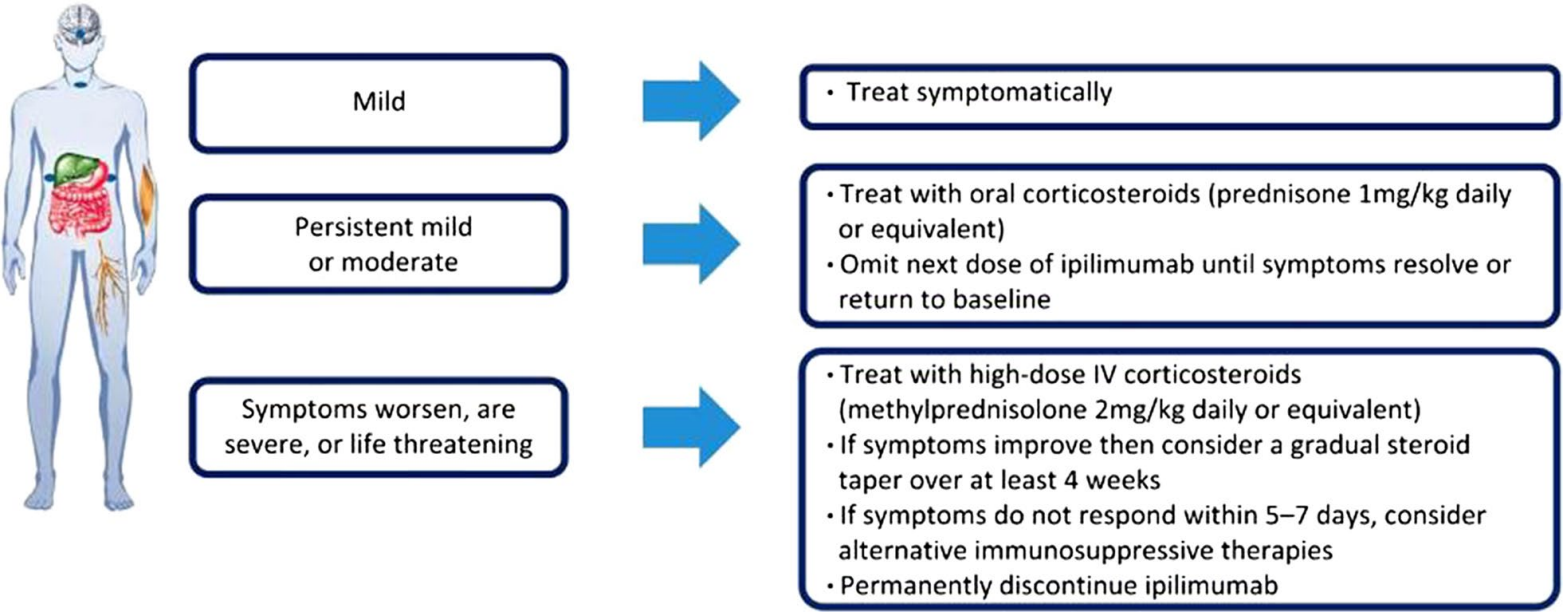
3-step approach in the treatment of irAEs in advanced melanoma with targeted therapies [18]

Treatment guidelines report that managing irAEs early enables completion of 12-week induction cycle. IrAEs are managed with product-specific treatment guideline and generally following a 3-step approach (Fig. 1).

Time to and durability of response in patients who discontinued due to toxicity was reported in previously untreated patients with unresectable stage III or IV melanoma to nivolumab alone, nivolumab plus ipilimumab, or ipilimumab alone [19]. They have been shown to have complementary activity in metastatic melanoma: a total of 38% of patients in the combination treatment discontinued due to toxicity, and 68% of them continued to respond even after the stopping of the treatment.

Besides, a statistically significant OS difference was noted in all patients (N = 143) in the combined cohorts who experienced any irAE versus those who did not (p = < 0.001), with greater OS benefit noted in patients who reported 3 or more irAE events (p = < 0.001) compared to those with none or only 1 irAE event [20].

Immunotherapy is also effective as adjuvant therapy (i.e. additional treatment given after the primary treatment for melanoma to reduce the risk of relapses) for patients with completely resected stage III melanoma at high risk of recurrence: median recurrence-free survival was 26.1 months (95% CI 19.3–39.3) in the ipilimumab group versus 17.1 months (95% CI 13.4–21.6) in the placebo group (HR 0.75; 95% CI 0.64–0.90; p = 0.0013); 3-year recurrence-free survival was 46.5% (95% CI 41.5–51.3) in the ipilimumab group versus 34.8% (30.1–39.5) in the placebo group [21]. The rate of OS at 5 years was 65.4% in the ipilimumab group, as compared with 54.4% in the placebo group (HR for death, 0.72; 95.1% CI 0.58–0.88; p = 0.001), and the rate of distant metastasis-free survival at 5 years was 48.3% in the ipilimumab group, as compared with 38.9% in the placebo group (HR for death or distant metastasis, 0.76; 95.8% CI 0.64–0.92; p = 0.002) [22]. Finally, the role of critical dosage is being addressed. The phase III CA 184–169 study directly compared two doses of ipilimumab in metastatic melanoma. Ipilimumab at 10 mg/kg every 3 weeks led to better overall survival (median = 15.7 months) than 3 mg/kg every 3 weeks (11.5 months; HR = 0.84; p = 0.04). Three-year survival rates were 31% versus 23%, respectively. Virtually all subgroups benefited from the 10 mg/kg dose, with the most impressive results seen for BRAF-positive patients (without prior treatment with a BRAF inhibitor), whose HR was 0.65. median PFS, however, was not improved with the higher dose, being 2.8 months in each arm; similarly, the objective response rate (15% vs. 12% for 10 mg/kg vs. 3 mg/kg, respectively) and disease control rate (32% vs. 28%) [16].

If the antibody-dependent cell cytotoxicity mechanism is more dose-dependent than other mechanism is still not clear: hypotheses have been proposed regarding an innate or adaptive resistance mechanism or a long-term sensitivity.

Finally, immunotherapy offers possibilities of combinations with other immunotherapies, target therapies, chemotherapy, radiotherapy to maximize the clinical benefit.

Among previously untreated patients with metastatic melanoma, nivolumab combined with ipilimumab resulted in significantly longer PFS than ipilimumab alone, with no new safety signals or drug-related deaths observed with the combination [17, 19].

The research should go further in exploring better combination with less side effects, the right duration of treatments, combination or sequencing treatments with target therapies.

**Fig. 2:**
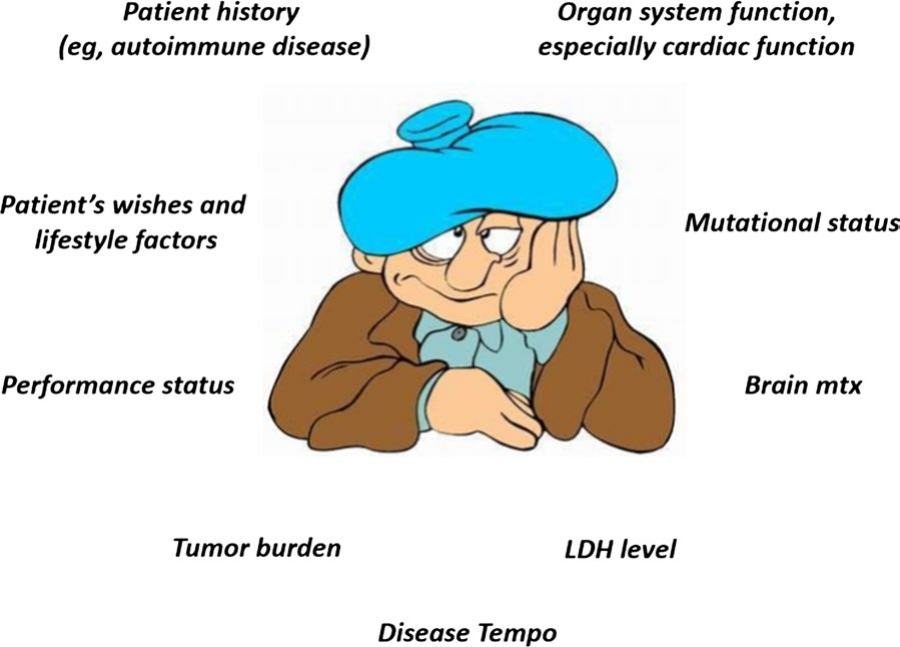
Main factors influencing treatment decisions

At present, treatment decision must be based on patient’s characteristic: disease history (e.g., autoimmune disease), performance status, tumor burden, organ system function, especially cardiac function, patients’ preferences and lifestyle factors, LDH level, mutational status (Fig. 2).

## Checkpoint inhibitors anti PD1/PDL1 in metastatic NSCLC

Great advance has been achieved in lung cancer like with EGFR activating mutation (erlotinib, gefinitib and afanitib or simertinib), ALK traslocation (crizotinib, ceritinib and alectinib), EGFR WT/ALK non squamous/squamous and platinum-based chemotherapies alone or in combination with bevacizumab. EGFR TKIs increased the median OS from 8–14 to 20–30 months with EGFR, ALK targeted treatments.

To date, two programmed death-1 inhibitors, namely nivolumab and pembrolizumab, have received the US FDA approval for the treatment of advanced NSCLC that failed platinum-based chemotherapy.

Several PD-L1 (atezolizumab, durvalumab, avelumab) and PD-1 (nivolumab, pembrolizumab, pidilizumab) inhibitors are currently under development. Phase I, Phase II, Phase III and concurrent clinical trials in 2–3 line for advanced disease versus adjuvant setting are ongoing.

## Monotherapy in pre-treated NSCLC: second-line of advanced NSCLC

Nivolumab is indicated for metastatic squamous-cell NSCLC with progression on or after platinum-based chemotherapy (CheckMate-017) [23]. It is the first PD-1 inhibitor to demonstrate survival benefit versus standard of care docetaxel in previously treated patients: 41% reduction in risk of death (HR 0.59, p = 0.00025), 1-year OS equal to 42% versus 24% and median OS equal to 9.2 months versus 6.0 [23]. Nivolumab demonstrated superiority over docetaxel also in all secondary end-points: the response rate was 20% with nivolumab versus 9% with docetaxel (p = 0.008); the median PFS was 3.5 months with nivolumab versus 2.8 months with docetaxel (HR for death or disease progression, 0.62; 95% CI 0.47–0.81; p < 0.001) [23]. Its benefit was independent of expression of the PD-1 ligand (PD-L1). Severe toxicity occurred less frequently with nivolumab (7% vs. 55%).

Nivolumab demonstrated superior OS versus docetaxel in patients with advanced non-squamous NSCLC after failure of platinum-based doublet chemotherapy: 27% reduction in risk of death (HR = 0.73. p = 0.0015). PD-L1 is predictive of benefit, started at the lowest expression level (1%): median OS nearly doubled with nivolumab versus docetaxel and no differences in OS were observed when PDL1 was not expressed in the tumor. Response Rate (ORR) nearly tripled in PDL1 expresser [24]. Nivolumab safety profile was favorable versus docetaxel and consistent with prior studies. Nivolumab also demonstrated superior OS (median OS 12.2 months in nivolumab group vs. 9.4 months in docetaxel group) and median PFS (2.3 months in nivolumab group vs. 4.2 months in docetaxel group), in patients with advanced non-squamous NSCLC after failure of platinum-based doublet chemotherapy and tyrosine kinase inhibitor [25].

In the KEYNOTE-010 study, the efficacy of pembrolizumab for patients with previously treated, PD-L1-positive, advanced NSCLC was assessed: pembrolizumab prolonged OS and had a favorable benefit-to-risk profile in patients with previously treated, PD-L1-positive, advanced NSCLC, introducing pembrolizumab as a new treatment option for this population and validate the use of PD-L1 selection [26]. In the total population, mOS was 10.4 months with pembrolizumab 2 mg/kg, 12.7 months with pembrolizumab 10 mg/kg and 8.5 months with docetaxel [26]. OS was significantly longer for pembrolizumab 2 mg/kg versus docetaxel (HR 0.71, 95% CI 0.58–0.88; p = 0.0008) and for pembrolizumab 10 mg/kg versus docetaxel (0.61, 0.49–0.75; p < 0. 0001) [26]. No significant difference in PFS for pembrolizumab 2 mg/kg versus docetaxel (0.88, 0.74–1.05; p = 0.07) or for pembrolizumab 10 mg/kg versus docetaxel (HR 0.79, 95% CI 0.66–0.94; p = 0.004) [26]. Among patients with at least 50% of tumor cells expressing PD-L1, OS was significantly longer with pembrolizumab 2 mg/kg than with docetaxel (median 14.9 months vs. 8.2 months; HR 0.54, 95% CI 0.38–0.77; p = 0.0002) and with pembrolizumab 10 mg/kg than with docetaxel (17.3 months vs. 8.2 months; 0.50, 0.36–0.70; p < 0.0001). Grade 3–5 treatment-related adverse events were less common with pembrolizumab than with docetaxel [26].

Atezolizumab (anti-PD-L1) is an engineered mAb that inhibits the PD-L1/PD-1 and PD-L1/B7. OAK data represent the first Phase 3 study results for a PD-L1-directed antibody. Atezolizumab improved OS in all patients: median 13.8 versus 9.6 months (HR 0.73); OS benefit was seen regardless of PD-L1 expression levels (HR 0.75 in < 1% PD-L1 expression population; 0.41 in ≥ 50% TC or ≥ 10% CI expression population); OS benefit was consistent across subgroups, including different histology (HR 0.73 for both), patients with CNS metastases (HR 0.54) and never smokers (HR 0.71) [27]. Atezolizumab was well tolerated with a favorable safety profile compared to docetaxel; no new safety signals were identified; the rate of immune-mediated AEs was low [27].

A recent meta-analysis assessed the role of immune checkpoint inhibitors as second-line therapy in EGFR-mutant advanced NSCLC [28]. The three included studies compared nivolumab [n = 292], pembrolizumab [n = 691] and atezolizumab [n = 144] against docetaxel (n = 776): the immune checkpoint inhibitors significantly prolonged OS over docetaxel alone (n = 1903, HR = 0.68, 95% CI 0.61–0.77, p < 0.0001) and in the EGFR wild-type subgroup (n = 1362, HR = 0.66, 95% CI: 0.58–0.76, p < 0.0001) but not in the EGFR-mutant subgroup (n = 186, HR = 1.05, 95% CI 0.70–1.55, p < 0.81; treatment-mutation interaction p = 0.03). Likely, mechanisms of acquired resistance to first-line tyrosine kinase inhibitor therapy should be elucidated to guide selection of second-line treatment for these patients.

## First line monotherapy and combination therapy in metastatic NSCLC

Nivolumab represents a standard of care in the second-line treatment of advanced NSCLC and in the first line setting nivolumab showed a promising response rate in a phase I trial in advanced NSCLC patients with 1% or greater PD-L1 expression in their tumour cells. However, greater patient selection may be needed for first line nivolumab to improve PFS over chemotherapy in advanced lung cancer, as the CheckMate 026 trial gave negative results in a broad group of patients expressing PD-L1 in their tumour cells.

In fact, the phase III CheckMate 026 trial investigated the efficacy of first line treatment with nivolumab compared to platinum-based doublet chemotherapy in patients with advanced NSCLC and PD-L1 positive tumours (defined as present in 1% or more tumour cells) [29]. In the 423 patients with 5% or greater PD-L1 expression, PFS was 4.2 months with nivolumab and 5.9 months with chemotherapy (HR 1.15, 95% CI 0.91–1.45, p = 0.25) [29]. OS was 14.4 months for nivolumab versus 13.2 months for chemotherapy (HR 1.02, 95% CI 0.80–1.30). Among all treated patients, any and serious treatment-related adverse events were 71 and 18% with nivolumab, and 92 and 51% with chemotherapy, respectively [29]. Nivolumab did not meet the primary endpoint of superior PFS compared with chemotherapy but OS was similar in the nivolumab and chemotherapy arms, and both compared favorably with historical controls (60.4% of patients in the chemotherapy arm received subsequent nivolumab); safety results were consistent with the known safety profile of nivolumab; there were fewer treatment-related grade 3–4 adverse events in the nivolumab versus chemotherapy arm.

A very relevant phase III trial (KEYNOTE 024) explored pembrolizumab as first line treatment compared to standard of care with platinum-based chemotherapy in untreated patients with advanced NSCLC and high PD-L1 expression (defined as expression in at least 50% of tumour cells) [30]. Patients with EGFR activating mutations and ALK translocations were excluded from recruitment to find better options than chemotherapy for these patients [30]. Patients in the chemotherapy arm who progressed were eligible to crossover to pembrolizumab as second line treatment (44% of these patients). The investigators found that pembrolizumab significantly improved the primary endpoint PFS by approximately 4 months compared to chemotherapy (10.3 months vs. 6.0 months, HR 0.50). The secondary endpoint OS was also significantly prolonged, and 80% of patients on pembrolizumab were alive at six months compared to 72% on chemotherapy (HR = 0.60). The significant improvement in OS with pembrolizumab was remarkable given that more than 40% of patients crossed over from the control arm to pembrolizumab after progression of the disease [30]. Pembrolizumab was associated with a higher overall response rate compared to chemotherapy (45% vs. 28%), a longer duration of response, and lower incidences of all and serious (3/4) adverse events.

Immunotherapies may show a benefit as first line treatment. Pembrolizumab in combination with carboplatin and pemetrexed is superior to carboplatin and pemetrexed alone as first-line therapy for advanced non-squamous NSCLC: ORR nearly doubled by adding pembrolizumab to chemotherapy: 55% versus 29% [31]. Risk of progression or death nearly halved: HR of 0.53 for PFS, with median PFS for pembrolizumab + chemotherapy exceeding 1 year. Similar OS between arms: 92% survival at 6 months in both arms [31]. The combination of pembrolizumab + carboplatin and pemetrexed is tolerable and has a manageable safety profile [31].

In the Phase 1 CheckMate 012 study nivolumab plus ipilimumab is well tolerated: frequency of treatment-related AEs leading to discontinuation was similar to nivolumab monotherapy (11–13%) [32]. There were no treatment-related deaths. Nivolumab plus ipilimumab has promising efficacy (39–47% ORR); median duration of response was not reached. Efficacy with nivolumab plus ipilimumab is enhanced with increasing PD-L1 expression:

- ≥ 1% tumor PD-L1 expression: 57% ORR; 83–90% 1-year OS rates.
- ≥ 50% tumor PD-L1 expression: 92% ORR [32].

Nivolumab 3 mg/kg Q2W plus ipilimumab 1 mg/kg Q6W schedule is being evaluated in further studies, including the ongoing phase III CheckMate 227 trial evaluates nivolumab monotherapy and nivolumab plus ipilimumab combination regimens versus PT-DC in patients with chemotherapy-naïve stage IV or recurrent squamous and non-squamous NSCLC [33].

No PD-L1 inhibitors have yet been approved for the treatment of NSCLC. Atezolizumab (MPDL3280A), durvalumab (MEDI4736), and avelumab are among the most advanced in clinical development.

A phase III trial with Atezolizumab in NSCLC as single agent in 1-line and combined to chemotherapy is ongoing.

Finally, neoadjuvant immunotherapy (i.e. additional treatment given before the main treatment, usually surgery) with the PD-1 inhibitor nivolumab was assessed as safe and feasible prior to surgery for early lung cancer, in patients with untreated, resectable, stage I-IIIA NSCLC underwent pretreatment tumor biopsy and then received two doses of nivolumab [34].

Survival benefit with PD-1/PD-L1 checkpoint inhibitors is independent of histologic subtype. However, some unsolved questions need answers: duration of therapy (1, 2 years, until progression of disease), use of single agent or combinations (chemotherapy, targeted therapies, other immunotherapy i.e. nivolumab + ipilimumab, pembrolizumab + ipilimumab, others), treatment strategy (upfront, maintenance), potential role in stage III, potential role in adjuvant setting.

Further research on the role of immune checkpoint inhibitors in subgroups such as EGFR/ALK-positive and current/former smokers is warranted. Further investigation of PD-L1 as a predictive biomarker is also required: PD-L1 appears predictive of response to PD-1/PD-L1 inhibitors in some settings; PD-L1 low/no expressing patients can still benefit from treatment. Continued research to identify potential biomarkers beyond PD-L1 is needed, for example smoking status, mutational landscape, other immune parameters, like tumor-infiltrating immune cells, immune-gene signatures, and ‘Immunoscore’.

A clinical consensus about several aspects is highly required: harmonization of all different IHC PD-L1 expression tests, new lab predictive factors (including results from liquid biopsy), treatment duration (i.e. until progression disease versus definite number of cycles), role in first-line as single agent and combined to chemotherapy, role in maintenance strategy, combination of immunotherapies, trials on Medium level of priority in the research can be given to studies on predictive role of IHC (produced by DAKO) PD-L1 expression for all anti PD-1 and anti PD-L1 inhibitors CT scan at definite time versus CT scan at clinically indication, neoadjuvant setting and combination with targeted therapies. Smokers plus former smokers versus never smokers can be targeted as low priority research.

## Head and neck cancer

The term head and neck carcinoma encompasses all malignancies arising in the nasal and oral cavities, pharynx, larynx and the paranasal sinuses. Majority of these (approximately 95%) epithelial cancers are squamous cell carcinomas. Head and neck squamous cell carcinoma (HNSCCs) are characterized by phenotypic, etiological, biological and clinical heterogeneity and can originate from the paranasal sinuses, nasal cavity, oral cavity, pharynx and larynx. The major known risk factors of HNSCC are consumption of tobacco and alcohol, as well as human papillomaviruses infection. Multiple studies have elucidated the specific genetic background of HNSCC, establishing subclasses of tumors alongside HPV infection and/or TP53 mutations. Tumor characteristics vary between patients of different ages.

Prognostic biomarker indicates the likely course of the disease in untreated patients (or regardless of treatment); predictive biomarker identifies subpopulations of patients who are most likely to respond to a given therapy. A variety of biomarkers have been reported in literature with a promising potential but these are still in the need of clinical validation. Table 2 reports the list of current biomarkers validated in the head and neck cancer.

**Table 2:** Biomarkers in the head and neck carcinoma

Biomarkers	Prognostic	Predictive
Epstein barr virus in endemic nasopharyngeal cancers	Validated	Validated
HPV	Validated	Potential
PET imaging post treatment	Validated	Potential
Hypoxia	Potential	Potential
EGFR (potential predictive factor for accelerated radiotherapy)	Potential	Not validated
TP53 gene mutation	Potential	Potential
Gene expression profiling	Potential	Potential
Immune checkpoint related	Potential	Potential

Early detection in head and neck cancer has been shown to dramatically increase survival rates when compared to detection at later disease stages, being the most important variable leading to positive outcomes [35]. Moreover, there are few screening tools and markers to discriminate the patients who are to be benefited by adjuvant therapy. Among biomarkers, HPV, especially HPV16, is considered one of the causing factors for HNSCC. HPV DNA has been found in 15–25% of HNSCC and the association differs depending on the site of the tumor; HPV DNA is detected in 45–67% of cases of cancers of the tonsil, in 13–25% of hypopharyngeal cancer, in 12–18% of the cancers of oral cavity and in 3–7% of carcinoma larynx and it may be associated with prognosis of disease, especially in tonsillar cancers.

Recurrent and/or metastatic HNSCC treatment options include chemotherapy or immunotherapy, re-irradiation, salvage surgery, best supportive care. Development of effective therapies for metastatic HNSCC has been challenging. Since the 80s when methotrexate and combination of cispaltinum and fluororuracil were used, new treatments arrived only in 2006 with cetuximab and in 2014–2015 with the approval of PD-1 inhibitors for solid tumors.

Tumor progression depends on acquisition of traits that allow cancer cells to evade immune surveillance and an effective immune response. HNSCC is an immunosuppressive disease, with lower absolute lymphocyte counts than those found in healthy subjects, impaired natural killer cell activity, and poor antigen-presenting function, impairment of tumor-infiltrating T lymphocytes [36]. In addition, suppressive regulatory T cells (Tregs) have been linked to HNSCC tumor progression. Tregs secrete suppressive cytokines such as TGFβ and IL-10, express CTLA-4 and correlate with tumor progression. Therefore, immunomodulatory therapies that overcome immune suppressive signals in patients with HNSCC have therapeutic promise. The FDA recently approved moAbs targeting immune checkpoint receptors, including anti-CTLA-4 and anti-PD-1, hopefully increasing the patients’ benefit from immunomodulatory therapies.

Only 3 studies have been currently completed and reported.

Nivolumab resulted in longer OS than treatment with standard, as single-agent therapy in patients with platinum-refractory, recurrent HNSCC [37]. In the CheckMate 141 study, an open-label, phase 3 trial (N = 361), the median OS was 7.5 months (95% CI 5.5–9.1) in the nivolumab group versus 5.1 months (95% CI 4.0–6.0) in the group that received standard therapy. OS was significantly longer with nivolumab than with standard therapy (HR for death, 0.70; 97.73% CI 0.51–0.96; p = 0.01), and the estimates of the 1-year survival rate were approximately 19% higher with nivolumab than with standard therapy (36.0% vs. 16.6%). The median PFS was 2.0 months (95% CI 1.9–2.1) with nivolumab versus 2.3 months (95% CI 1.9–3.1) with standard therapy (HR for disease progression or death, 0.89; 95% CI 0.70–1.13; p = 0.32). The rate of PFS at 6 months was 19.7% with nivolumab versus 9.9% with standard therapy. The response rate was 13.3% in the nivolumab group versus 5.8% in the standard-therapy group. Treatment-related adverse events of grade 3 or 4 occurred in 13.1% of the patients in the nivolumab group versus 35.1% of those in the standard-therapy group. Physical, role, and social functioning was stable in the nivolumab group, whereas it was meaningfully worse in the standard-therapy group [37].

Pembrolizumab was well tolerated and demonstrated clinically meaningful antitumour activity in recurrent or metastatic HNSCC, PD-L1 expression [38]. The proportion of patients (N = 104) with an overall response by central imaging review was 18% (95% CI 8–32) in all patient, it was 25% (95% CI 7–52) in HPV-positive patients and 14% (95% CI 4–32) in HPV-negative patients. Pembrolizumab was well tolerated, with 17% of patients having grade 3–4 drug-related adverse events and 45% experiencing a serious adverse event [38].

The preliminary results from the phase 2, non-randomized KEYNOTE-055 study evaluating pembrolizumab after progression on platinum and cetuximab in recurrent or metastatic HNSCC, showed that, on the first 50 patients enrolled (median follow-up time was 6.8 months), 12% patients experienced grade 3-5 treatment-related adverse events; the stable disease rate was 18.0% [39].

Several anti PD1 and vaccine studies are currently ongoing on HNSCC. We just want to mention the phase III studies regarding the anti-PD1 and currently recruiting. A first line study (Keynote 48) with, pembrolizumab versus pembrolizumab + platinum/5-FU versus Cetuximab + Platinum/5-FU is ongoing. In recurrent/metastatic NHSCC patients after 6-month curative therapy; PFS was the primary endpoint. A further study, including anti-PD1 and antiCTL4 regarded durvalumab versus durvalumab + tremelimumab versus cetuximab/platinum/5-FU (KESTREL study); PFS and OS were the end-pointsAnti-PD-1 pembrolizumab versus cetuximab, methotrexate or docetaxel is under evaluation in platinum refractory/metastatic HNSCC (EAGLE study)Durvalumab versus durvalumab + tremelimumab versus standard of care in platinum refractory R/M HNSCC < 6 months from therapy containing platinum PD-L1+ is also ongoing.

## Kidney and prostate cancer

Prior to the advances in therapeutics seen over the last decade, the mainstay of treatment for metastatic kidney disease was cytokine-based treatment with high dose interleukin-2 (IL-2) and interferon-alpha (IFN-α) after their FDA approval in the 1990s. Although this therapy regimen produced objective responses, there were significant toxicities, treatment benefit was only seen in 5–15% of patients, and outcome for most patients was poor. Since 2004, the advances in target-based therapy and immunotherapy modalities have created a paradigm shift in the treatment of renal carcinoma. These agents have had a remarkable effect on patient outcomes with increased PFS rates; however, virtually all patients eventually progress. The high likelihood of disease progression remains a challenge due to therapeutic resistance. Refractory disease is currently being managed with sequentially changing therapy, but morbidity and mortality remain high.

The agents approved for the first-line treatment of metastatic renal carcinoma has rapidly developed over the years and now includes the small-molecule VEGF Tyrosine Kinase Inhibitor (TKI)-sunitinib and pazopanib, a monoclonal antibody targeting VEGF-bevacizumab in combination with interferon, and a mammalian target of rapamycin (mTOR) inhibitor-temsirolimus, as well as high dose IL-2. In the recent past, the approach to the treatment of patients with mRCC entailed sequential employment of agents targeting VEGF or mTOR pathways. Agents with anti-angiogenesis properties have become the mainstay of initial therapy for advanced renal carcinoma due to their preferable efficacy and toxicity profile. The current level 1 recommendation from ESMO is the use of oral, multi-target, TKIs—specifically sunitinib and pazopanib—in the first-line setting (Table 3) [40].

**Table 3:** ESMO 2014 treatment guidelines—recommended treatments based on risk stratification [40]

Setting	Treatment group	Standard	Option
First line	Good or intermediate risk	Sutinib [I, A] Bevacizumab + IFN-α [I, A] Pazopanib [I, A]	High-dose IL-2 [III, C] Sorafenib [II, B] Bevacizumab + low-dose IFN-α [III, B]
	Poor risk	Temsirolimus [II, A]	Sutinib [II, B] Sorafenib [III, B] Pazopanib [III, B]
Second line	Post cytokines	Axitinib [I, A] Sorafenib [I, A] Pazopanib [II, A]	Sutinib [III, A]
	Post-TKI	Nivolumab [I, A] Cabozantinib [I, A]	Axitinib [II, B] Everolimus [II, A] Sorafenib [III, B]
Third line	Post-two VEGF-TKIs	Nivolumab [II, A] Cabozantinib [II, A]	Everolimus [II, B]
	Post TKI and mTOR	Sorafenib [I, B] Nivolumab [V, A] Cabozantinib [V, A]	Other TKI [IV, B] Rechallenge [IV, B]
	Post TKI/nivolumab	Cabozantinib [V, A]	Axitinib [IV, C] Everolimus [IV, C]
	Post TKI/cabozantinib	Nivolumab [V, A]	Axitinib [V, C] Everolimus [V, C]

Metastatic renal carcinoma is highly immunogenic. Immunotherapy involves the activation of endogenous immune system to target cancer at cellular level and enable checkpoint inhibition in two-principle immune signaling mechanisms: CTLA-4 and PD-1. Anti-PD1/PDL1 is the backbone of future combination immunotherapies and anti-PD1/PDL1 combination therapies are expected to disrupt the current treatment paradigms in kidney cancer.

From a study on 821 patients with advanced clear renal carcinoma, receiving previous treatment with one or two regimens of antiangiogenic therapy receiving nivolumab or everolimus, the OS was 25.0 months (95% CI 21.8 to not estimable) with nivolumab and 19.6 months (95% CI 17.6–23.1) with everolimus, with an HR for death with nivolumab versus everolimus of 0.73 (98.5% CI 0.5–0.93; p = 0.002). The median PFS was 4.6 months (95% CI 3.7–5.4) with nivolumab and 4.4 months (95% CI 3.–5.5) with everolimus (HR, 0.88; 95% CI 0.75–1.03; p = 0.11) [41] (CheckMate 025 study).

Studies on combinations and immunotherapy in first line are ongoing. Nivolumab in combination with ipilumumab showed acceptable safety and encouraging antitumor activity in metastatic renal carcinoma with most responses ongoing [42]. More recently, the CheckMate 214 was designed as phase 3 study of Nivolumab + Ipilimumab versus Sunitinib in previously untreated mRCC, with then Nivolumab 3 mg/kg solutions until documented disease progression, discontinuation due to toxicity, withdrawal of consent or the study ends [43]. A phase II study on atezolizumab, an engineered anti-PD-L1 Antibody as monotherapy or in combination with bevacizumab compared to sunitinib in untreated advanced RCC [44] has failed to demonstrate an improved PFS over sunitinib, but shows promise PDL1 positive subsets. The phase III trial is still ongoing.

Updated ESMO renal cancer 2016 guidelines reported no current evidence that new checkpoint inhibitors should be used in first line, although numerous ongoing trials are exploring their role, either as monotherapy or in combination (with either VEGF inhibitors or other checkpoint inhibitors), mostly on cabozantinib. Second-line treatments have recently been dramatically modified by the report of two large trials showing improvement in OS with nivolumab and cabozantinib over everolimus: both trials showed very significant improvement in OS and response rate, while PFS was improved only in the cabozantinib trial. In both trials, patients could be treated after either one or two TKIs [41, 45, 46].

A further management strategy is conservation. Because of the toxicity and non-curative nature of current systemic therapy, selected patients may benefit from initial surveillance only. Metastatic renal cancer patients can be safely observed for a period before starting systemic therapy. This was demonstrated in a prospective phase II observation trial in pts with mRCC prior to initial systemic treatment. Median months of observation until start of systemic therapy was 14.1 months with periodic CT assessment. Initiation of systemic treatment was discretionary, according to tumor size, location or number of metastases [47].

Nevertheless, how long should be the schedule and length of treatment, which other combination can be investigated, Other Immune Checkpoints (CTLA4,LAG3, Kir,..) use, agonist (Ox40, GITR..) and vaccines use, TME modifiers (VEGF TKI, MSDC/Treg depletion, IDO Inhibitors) and adoptive/CAR T-cell therapy remain open. Finally, several trials evaluating PD1 and PDL1 inhibitors in the adjuvant space are accruing.

## Immunotherapy in advanced hepatocarcinoma

Advanced Hepatocarcinoma (HCC) (Barcelona Clinic Liver Cancer, BCLC stage C or stage B no longer suitable for locoregional treatments) patients with cancer related symptoms (symptomatic tumors, ECOG 1–2), macrovascular invasion (either segmental or portal invasion) or extrahepatic spread (lymph node involvement or metastases) bear a dismal prognosis, with expected median survival times of 7–8 months or 25% at 1 year. In 2006, there was no approved first line treatment for patients with advanced HCC. This scenario changed because of data showing survival benefits in patients receiving sorafenib—a multi tyrosine kinase inhibitor—in patients with HCC not eligible for surgery or locoregional treatments. These results represented a breakthrough in the management of HCC: median OS was significantly longer in the sorafenib group than in the placebo group (10.7 months vs. 7.9 months; HR in the sorafenib group, 0.69; 95% CI 0.55–0.87; p < 0.001). At 1-year sorafenib provided significant survival benefit representing a 31% relative reduction in the risk of death. Sorafenib resulted effective and well tolerated for the treatment of advanced HCC also in patients from the Asia–Pacific region: median OS was 6.5 months (95% CI 5.56–7.56) in patients treated with sorafenib, compared with 4.2 months (3.75–5.46) in those who received placebo (HR 0.68 95% CI 0.50–0.93; p = 0.014) [48].

Based on promising activity in their second-line phase 2 study Bruix et al. evaluated regorafenib, an oral multikinase inhibitor, in patients with intermediate or advanced HCC who had disease progression on sorafenib [49]. Adults with HCC BCLC stage B or C received sorafenib until radiological progression and were then randomized to regorafenib. The regorafenib group had a 38% reduction in the risk of death (HR 0.62; 95% CI 0.50‒0.78; p < 0.001); median OS (regorafenib vs. placebo) was 10.6 versus 7.8 months; there was a 54% reduction in the risk of progression or death with regorafenib (HR 0.46; 95% CI 0.37‒0.56; p < 0.001); median PFS (regorafenib vs. placebo) was 3.1 versus 1.5 months [49]. Based on these results, very recently the FDA expanded the indications of regorafenib to include the treatment of patients with HCC previously treated with sorafenib.

The last 9 years have seen novel therapeutic contenders struggle to improve outcomes and remove sorafenib as front-line therapy. This is due, in part, to liver dysfunction (cirrhosis) shown in many HCC patients as well as other comorbidities resulting from infection with hepatitis B or hepatitis C, and/or occurrence of non-alcoholic fatty liver disease. Noticeably, systemic therapies such as sorafenib are limited to patients with a good liver function (Child–Pugh A). Patients with impaired liver function (Child–Pugh B or C) may not tolerate current therapeutic options and generally receive only best supportive care. Even those with reasonable liver function may struggle to tolerate combination therapies that include sorafenib as a backbone. Thus there is a great need of a novel efficient and tolerable agent to include more patients.

Hepatocarcinogenesis is a multistep process depending on a sequence of epigenetic and genetic alterations leading to activation or inhibition of p53, WNT, β-catenin, MYC, the ErbB family, as well as chromatin modifications. Unlike other solid tumors, the specific sequence of genetic events that mediate hepatocarcinogenesis is not known. HCC development depends on mutations in approximately 140 genes belonging to 12 signalling pathways regulating cell fate, cell survival, and genome maintenance. HCC usually progresses from chronic hepatitis, to cirrhosis, to dysplastic nodules (low- and high-grade), and finally to malignant tumors. Gene expression studies identified MYC and TLRs as important mediators of malignancy. Nevertheless, specific genetic variants were not associated with HCC [50]. Personalized medicine in HCC needs first to identify and validate molecules required for HCC growth or progression and develop specific inhibitors of these factors. For example, WNT and RAS are activated in 25 and ~ 50% of HCCs, respectively, but specific inhibitors have not entered trials for HCC. HCCs have significant amounts of genomic heterogeneity and multiple oncogenic pathways can be activated. Studies are needed on large numbers of tumor specimens, to identify the best therapeutic targets [50].

Pathogenesis and the survival of patients with HCC impacts the failure of the immune system to prevent HCC and to halt its progression. Thus, immunotherapy aiming at increasing HCC-specific immune responses is considered a promising treatment approach. HCC is typically an inflammation-associated cancer and can be immunogenic [51]. Besides, the association of hepatitis C and hepatitis B infection with upregulation of PD-1 has also been demonstrated, so that PD-1 expression could be utilized as a potential clinical indicator to determine the extent of virus replication and liver injury [52].

Upregulation of PD-1 and the PD-1 immune checkpoint ligand, PD-L1, in HCC is associated with poor outcomes: circulating PD-1/PD-L1 expression was associated with severity of diseases in patients with HCC; moreover, PD-1/PD-L1 expression was associated with clinical parameters as tumor size blood vessel invasion and BCLC staging [53]. Patients with higher expression of circulating PD-L1, as well as circulating PD-1, had a significantly shorter OS and tumor-free survival than those with lower expression. A multivariate analysis confirmed that circulating PD-L1 could serve as an independent predictor of OS and tumor-recurrence survival in HCC patients after cryoablation [53]. Blockade of PD-1 with monoclonal antibodies combined with immunostimulatory monoclonal antibodies extended survival [54, 55]; immune checkpoint inhibition (anti-CTLA-4) has shown encouraging activity in an early clinical trial in HCC [56].

Nivolumab in advanced HCC showed durable OR, irrespective of viral infection (HBV, HCV), increasing OS rates, maintaining a good safety and RR not correlated with tumor PD-L1 expression [57].

The CheckMate-040 Phase I/II trial was initiated to evaluate the safety, tolerability, dose-limiting toxicities, and maximum tolerated dose of nivolumab in (1) uninfected HCC subjects, (2) HCC patients with hepatitis B, and (3) HCC patients with hepatitis C. Patients had advanced HCC, with a Child–Pugh score of ≤ 7 (dose escalation) or ≤ 6 (dose expansion). Exclusion criteria was active HBV infection and patients with a viral load were required to be on antiviral therapy with a viral load of < 100 IU/mL. Subpopulations of patients were nivolumab-naïve and nivolumab-refractory [57]. In October 2016, interim data were presented for 48 patients treated in the dose escalation cohort and 214 patients in the dose-expansion cohort [57]. 25% of patients experienced Grade 3/4 or greater adverse events; hematologic liver parameters were considered manageable and did not result in hepatitis. EQ-5D index scores did not show differences in first- or second-line patients and were stable from baseline to week 25. Thus, the relatively mild toxicity profile of nivolumab monotherapy in these pretreated HCC patients was encouraging.

In second-line nivolumab-experienced patients, 37 patients were evaluable in the escalation cohort and 145 were evaluable in the expansion cohort. ORR were 16.2 and 18.6% in the escalation and expansion phases, respectively, including Complete Response (CR) rates of 8.1 and 2.1%. The duration of response was 17.1 months in the dose escalation cohort and had not been reached in the dose expansion cohort. The median OS of the dose escalation cohort was 15.0 months; it was 13.2 months in the dose expansion cohort. At 9 months’ follow-up, 67 and 71% of patients were alive in the escalation and expansion cohorts, respectively. In the escalation cohort, 46% of patients were alive at 18 months. In the dose expansion nivolumab-naïve cohort (69 patients), 21.7% had an objective response (all partial). 6- and 9-month OS rates were 87 and 77%, respectively. Expression of PD-L1 did not correlate with response to nivolumab in either patient population [57].

Results from the CheckMate-040 trial were very promising. Based on these encouraging results, in November 2015, a randomized global Phase III head-to-head trial (CheckMate-459) of nivolumab versus sorafenib initiated. The trial is recruiting treatment-naïve, Child–Pugh A advanced HCC patients in the United States, EU, Asia and Australia. Another ongoing Phase III trial (Keynote-240, NCT02702401) is investigating pembrolizumab versus best supportive care in relapsed/refractory HCC.

## Immunotherapy in ovarian cancer

Epithelial ovarian cancer (EOC) is the leading cause of death for gynecological cancer. Despite the recent introduction of new drugs in the therapeutic armamentarium (PARP inhibitors, antiangiogenic) the rate of recurrence is still high (70%) and overall prognosis remains globally severe. Ovarian cancer is considered an immunogenic tumor that can be recognized and attacked by the immune system [58]. The analysis of gene profiling of high grade serious ovarian cancer identified immunoreactive tumors associated to better prognosis. The Cancer Genome Atlas project has analysed has analyzed the DNA sequences of exons IN 489 high-grade serous ovarian adenocarcinomas from coding genes in 316 of these tumors and reported that high-grade serous ovarian cancer is characterized by TP53 mutations in almost all tumors (96%) [59].

Although tumor-infiltrating T cells have been documented in ovarian carcinoma, a clear association with clinical outcome was not established until 2003: the five-year OS rate was 38% among patients whose tumors contained T cells and 4.5% among patients whose tumors contained no T cells in islets; after CR with chemotherapy, only patients with tumor-infiltrating lymphocytes (TIL) survive or are in remission long-term [58].

Sato et al. demonstrated that intraepithelial CD8+ tumor-infiltrating lymphocytes and a high CD8+/regulatory T cell ratio is associated with favorable prognosis in ovarian cancer: patients with higher frequencies of intraepithelial CD8+ T cells demonstrated improved survival compared with patients with lower frequencies (median: 55 vs. 26 months) [60]. A meta-analysis of studies (N = 1815 patients) evaluating the prognostic value of TIL on survival confirmed TILs are a robust predictor of outcome in ovarian cancer and define a specific class of patients [61].

Different histotypes are observed that looks like epithelial cells; two groups of epithelial ovarian cancers have been distinguished: type I low-grade cancers that present in early stage, grow slowly, and resist conventional chemotherapy but may respond to hormonal manipulation and type II high-grade cancers that are generally diagnosed in advanced stage and grow aggressively but respond to chemotherapy [62]. Type I cancers have wild-type p53 and BRCA1/2, but also mutations of Ras and Raf as well as expression of IGFR and phosphatidylinositol-3-kinase (PI3K) pathway; type II cancers have mutations of p53 and BRCA1/2 [62].

Others immune factors correlated with bad prognosis are presence of Treg in the tumor [63–66], accumulation of plasmacytoid dendritic cells [67–69] presence of immunosuppressive macrophages expressing B7-H4 [70], low level of circulating lymphocytes (< 1.0 × 109/L) [71].

The expression of PD-L1 in ovarian was explored since 2006: a significant inverse correlation was observed between PD-L1 expression and the intraepithelial CD8+ T lymphocyte count, suggesting that PD-L1 on tumor cells directly suppresses antitumor CD8+ T cells; the expression of PD-L1 on tumor cells and intraepithelial CD8+ T lymphocyte count are independent prognostic factors. Thus, the PD-1/PD-L pathway can be a good target for restoring antitumor immunity in ovarian cancer [72, 73].

Nivolumab showed to mediate tumor regression in a substantial proportion of patients with ovarian cancer in phase II trial and it currently also demonstrated durable anti-tumor response in patients with platinum-resistant ovarian cancer: complete response patients were alive (2 out of 20) without tumor relapse after they had completing the 1 year the trial [74].

Avelumab is a fully human anti-PD-L1 IgG1 antibody wth antitumor activity in bladder, lung, gastric and other malignancies as demonstrated in preclinical models. In patients previously treated, affected by recurrent or refractory ovarian cancer, single-agent avelumab showed an acceptable safety profile and clinical activity: overall, median PFS was 11.3 weeks (95% CI 6.1, 12.0) and median OS was 10.8 months (95% CI 7.0, 16.1) [75]. The potential relationship between biomarkers, such as germline BRCA mutational status, and the probability of response is under investigation. The phase III trial of Avelumab in combination with and/or following platinum-based chemotherapy, in patients previously untreated and affected by advanced epithelial ovarian, fallopian tube cancer, or primary peritoneal cancer candidates for platinum-based chemotherapy is currently ongoing (JAVALIN OVARIAN 100 Study). Its primary purpose is to demonstrate that avelumab as single agent efficacy in the maintenance setting, following frontline chemotherapy or in combination with carboplatin/paclitaxel, is superior to platinum-based chemotherapy alone NCT 02718417).

Since PD-L1 was found to be overexpressed in ovarian cancer and can contribute to malignancy. Pembrolizumab was initiated in patients with PD-L1+ advanced solid tumors and demonstrated that 23% of patients experienced a decrease in target lesion (KEYNOTE-028NCT02054806 phase Ib trial) demonstrated [12].

There is a lack of validated predictive biomarkers of response in EOC. Expression of PD-L1 on tumor cells is not a reliable predictive of benefit from immune checkpoint inhibitors. Heterogeneous techniques in the measurement of PD-L1 and different timing of assessments subgroups may benefit more from immune checkpoint inhibitors: somatic and germline BRCA mutation carriers [76]. BRCA1/2-mutated high grade serious ovarian cancer may be more sensitive to PD-1/PD-L1 inhibitors compared to HR-proficient high grade serious ovarian cancers. BRCA1/2-mutated tumors exhibited significantly increased CD3+ and CD8+ TILs and elevated expression of PD-1 and PD-L1 in tumor associated immune cells compared to HR-proficient tumors. Besides, BRCA1/2-mutation status and number of TILs were independently associated with positve outcome. HR proficient with low number of TILs group showed very poor prognosis and BRCA1/2-mutated tumors with high number of TILs group showed very good prognosis [76].

Furthermore, clear cell ovarian cancer is characterized by an intrinsic chemoresistance: studies of immune checkpoint inhibitors in ovarian cancer have demonstrated isolated responses in tumors with cell ovarian cancer histology. Cell ovarian cancers are frequently associated with MicroSatellite Instability (MSI) leading to a higher number of CD3+ TILs and PD-1+ TILs [76]. Cell ovarian cancers have a high rate of alterations in the PI3K/Akt/mTOR pathway, that correlates with an increased expression of PD-L1 in tumor cells in NSCLC [77].

Based on few clinical trials available, platinum-resistant EOCs, which are characterized by unfavorable prognosis and general chemoresistance, seem a reasonable target. Patients with lower tumor burden, which usually identifies platinum-sensitive disease, may be more favorable in terms of arming immune system against cancer.

Combine checkpoint inhibitors with other systemic therapy can increase clinical benefit. Platinum-derived compounds increase the release of TAAs and stimulate the immune response. Why this does not improve survival is on studying: the cytokine release syndrome has ongoing trials evaluating the addition of celecoxib or ASA to cisplatin and PD-L1 blockade.

Ovarian cancer is known to have an angiogenic phenotype: VEGF has an immune suppressive effect on T cells activation and inversely correlates with TILs infiltration: the association between checkpoint inhibitors and antiangiogenic drugs appears reasonable [78]. A phase III trial is ongoing to evaluate atezolizumab in combination with carboplatin–paclitaxel–bevacizumab in untreated patients with ovarian cancer (NCT03038100).

In conclusion, the treatment of advanced/relapsed EOC remain clearly an unmet need. In fact, immunecheckpoint inhibitors may improve clinical outcome, but before considdering them therapeutic options several questions need to be addressed: what are reliable predictors of response in EOC? Are there subgroups more likely to benefit from immune checkpoint inhibitors? Is it better to use checkpoint inhibitors alone or in association with other agents?

## Emerging role of immunotherapy in urothelial carcinoma

Urothelial carcinomas (UCs), also known as transitional cell carcinoma, is the most common histological subtype of carcinomas in the urinary tract. It arises from the urinary tract anywhere from renal pelvis, ureter, urethra and bladder. There are other, but much less common (~ 5%), histological subtypes of urinary tract cancer, including squamous cell carcinoma, adenocarcinoma, small cell carcinoma, and mixed histology of UC plus any of these.

In the United States, approximately 75,000 new cases of bladder cancer are estimated to be diagnosed in 2017, making it the fifth most common cancer among adults [79]. Worldwide, approximately 500,000 cases are diagnosed annually [80]. Several environmental risk factors such as cigarette smoking, occupational exposures, and infectious agent, have been identified. The presence of high rates of somatic mutations may enhance the ability of the host immune system to recognize tumour cells as foreign owing to an increased number of antigens. However, these cancers may also elude immune surveillance and eradication through the expression of programmed death-ligand 1 (PD-L1; also called CD274 or B7-H1) in the tumour microenvironment [81].

Cisplatin-containing combination chemotherapy with GC (gemcitabine/cisplatin), or MVAC (methotrexate, vinblastine, adriamycin and cisplatin) has been the standard systemic treatment for metastatic urothelial carcinoma for several decades [82–84]. GC has better safety profile and tolerability than MVAC [82]. MVAC is better tolerated with the use of granulocyte colony-stimulating factor (G-CSF) [83, 84]. In the United States, there has not been a standard systemic therapy for patients who progressed after platinum-based chemotherapy until the US FDA approval of atezolizumab in May 2016. In Europe, vinflunine, a third generation vinca alkaloid, is an EMA approved second-line therapy for patients who progressed after platinum-containing therapy. The approval was based on the data showing an improvement of median overall survival by 2.6 months (6.9 vs. 4.3 months; p = 0.036) in the eligible patients although the survival analysis in the intent-to-treat population did not meet the statistical significance [81].

A major limitation in systemic treatment of advanced UC is that approximately 30 to 50% of patients are medically unfit to receive the standard, cisplatin-containing combination chemotherapy [85, 86]. This is because UC is largely a disease of the elderly, hence patient have impaired renal function related their age and disease, and poor performance status. For patients unfit for cisplatin, therapeutic options are limited and survival is poor. Gemcitabine and carboplatin combination therapy is commonly used in this setting based on the EORTC study 30,986 showing 36% confirmed ORR and 9.3 months median OS. However, the regimen is still associated with significant toxicity with 21% having to discontinue treatment due to toxicity [87]. On this grim landscape, the emergence of FDA’s approved immunotherapy for treatment of metastatic UC represents a paradigm shift.

### First line

#### Atezolizumab

In April 2017, the FDA has granted an accelerated approval to atezolizumab as frontline treatment for cisplatin-ineligible patients with locally advanced or metastatic UC (mUC). This approval was based on data from the single-arm phase II IMvigor210 trial. In a study cohort of 119 cisplatin-ineligible, treatment-naive patients, the ORR with atezolizumab was 23% (n = 28; 95% CI 16–32), including a CR rate of 6.7% [88]. Median response duration was not reached. Responses occurred across all PD-L1 and poor prognostic factor subgroups. Median PFS was 2.7 months (2.1–4.2). Median overall survival was 15.9 months (10.4 to not estimable). Tumour mutation load was associated with response. Treatment-related adverse events that occurred in 10% or more of patients were fatigue (30%), diarrhoea (12%), and pruritus (11%). One treatment-related death (sepsis) occurred. Nine (8%) patients had an adverse event leading to treatment discontinuation. Immune-mediated events occurred in 14 (12%) patients.

There is an ongoing phase III study, IMVigor130 trial to study atezolizumab as monotherapy and in combination of platinum-based chemotherapy with or without atezolizumab (NCT02807636). Primary efficacy outcome measures are progression free survival by investigator, and overall survival.

#### Pembrolizumab

Clinical activity of pembrolizumab, anti-PD-1 antibody, was also evaluated in a phase II study, KEYNOTE052 trial in the same patient population. This was reported in 2017 GU ASCO by Balar et al. [89]. The study showed the ORR (95% CI) of 27% (22–32%) among pts with ≥ 4 month follow-up (n = 307) with CR of 6%. Among the ≥ 4 month follow-up group, median (range) time to response was 2.0 (1.6–4.8) month; median (range) duration of response was not reached (1 + to 14 + month). 78% of responders had a response for ≥ 6 month (KM estimate). PFS and OS rates at 6 month were 31 and 67%, respectively (KM estimate).

A phase III study, KEYNOTE-361 is also ongoing for patients with advanced, metastatic UC as frontline therapy. This study compares pembrolizumab monotherapy versus platinum-based chemotherapy with or without pembrolizumab in chemotherapy naive patients (NCT02853305). This study is powered for two co-primary endpoints, PFS using RECIST assessed by blinded independent central review and overall survival.

#### Durvalumab

Durvalumab, anti-PD-L1 antibody, is currently being evaluated in a randomized phase III trial, DANUBE. This study investigates the efficacy and safety of durvalumab as monotherapy and in combination with tremelimumab (ant-CTLA4 antibody) versus standard of care first-line chemotherapy in treatment naïve patients with stage IV urothelial carcinoma (NCT02516241).

### Second line

#### Atezolizumab

Atezolizumab marked the first-in-class immune checkpoint inhibitor approved for advanced urothelial carcinoma. As noted above, on May 18, 2016, atezolizumab was granted an accelerated approval for second-line treatment of patients with advanced urothelial carcinoma [90]. This approval was based on data of a single-arm trial in 310 patients with locally advanced or metastatic urothelial carcinoma who had disease progression after prior platinum-containing chemotherapy. Patients received atezolizumab 1200 mg intravenously every 3 weeks until disease progression or unacceptable toxicity. The primary efficacy measures were ORR by Independent Review per RECIST 1.1, and duration of response. With a median follow-up of 14.4 months, confirmed ORR was 14.8% (95% CI 11.1, 19.3) in all treated patients. Median duration of response was not reached and response durations ranged from 2.1 + to 13.8 + months. Of the 46 responders, 37 patients had ongoing response for ≥ 6 months. The most common adverse reactions (≥ 20%) were fatigue, decreased appetite, nausea, urinary tract infection, pyrexia, and constipation. Infection and immune-related adverse events also occurred, including pneumonitis, hepatitis, colitis, endocrine disorders, and rashes. A phase III trial, IMvigor211, is currently ongoing to compare atezolizumab versus investigator’s choice of chemotherapy which included vinflunine, paclitaxel or docetaxel in patients with previously treated metastatic urothelial carcinoma (NCT02302807). The primary outcome measure is overall survival. This study is intended to confirm the findings of the phase II IMvigor210 study.

#### Nivolumab

In February 2017, the FDA approved the second immune checkpoint inhibitor for bladder cancer. Nivolumab, anti-PD-1 antibody, was granted an accelerated approval by the US FDA for the treatment of patients with locally advanced or metastatic urothelial carcinoma whose disease has progressed during a period of up to 1 year after first-line platinum-containing chemotherapy. The approval was based on a single-arm study, CheckMate-275 study, in 270 patients with locally advanced or metastatic urothelial carcinoma who experienced disease progression during or following platinum-containing chemotherapy, or whose disease progressed within 12 months of neoadjuvant or adjuvant treatment with platinum-containing chemotherapy [91]. Patients received nivolumab, 3 mg/kg every 2 weeks, until disease progression or unacceptable toxicity. The objective response rate was 19.6% (53 of 270 patients; 95% confidence interval, 15.1–24.9). The estimated median duration of response was 10.3 months. Responses were confirmed by an independent radiographic review committee using Response Evaluation Criteria in Solid Tumors 1.1. Confirmed objective response was achieved in 23 (28.4%, 95% CI 18.9–39.5) of the 81 patients with PD-L1 expression of 5% or greater, 29 (23.8%, 95% CI 16.5–32.3) of the 122 patients with PD-L1 expression of 1% or greater, and 23 (16.1%, 95% CI 10.5–23.1) of the 143 patients with PD-L1 expression of less than 1%. The most common adverse reactions (reported in 20% or fewer patients) were fatigue, musculoskeletal pain, nausea, and decreased appetite. Fourteen patients died from causes other than disease progression. These patients included four who died from pneumonitis or cardiovascular failure attributed to nivolumab. Adverse reactions led to dose discontinuation in 17% of patients. The recommended dose and schedule for nivolumab for the above indication is 240 mg intravenously every 2 weeks.

The clinical activity of nivolumab was first studied in a phase I/II study, CheckMate-032 study as a monotherapy and in combination with ipilimumab [92]. Sharma et al. published the safety and clinical activity data of nivolumab as a monotherapy in 86 patients with metastatic urothelial carcinoma [93]. Patients received nivolumab 3 mg/kg IV every 2 weeks. A confirmed investigator-assessed objective response rate was 24.4% (95% CI 15.3–35.4). CheckMate032 study also evaluated clinical safety and activity of nivolumab in combination with ipilimumab. The preliminary data of the ongoing phase I/II study of CheckMate032 showed that nivolumab in combination with ipilimumab is active and well tolerated among patients with previously treated metastatic UC. The study evaluated two different doses of ipiliumab and nivolumab. The combination of nivolumab 1 mg/kg and ipilimumab 3 mg/kg showed an ORR of 38.5% (95% CI 20.2–59.4), whereas the doses of nivolumab 3/mg/kg plus ipiliumab1 mg/kg led to an ORR of 26.0% (95% CI 17.9–35.5).

#### Durvalumab

On May 1, 2017, durvalumab (IMFINZI, AstraZeneca UK Limited) was granted accelerated approval for the treatment of patients with locally advanced or metastatic urothelial carcinoma who have disease progression during or following platinum-containing chemotherapy or who have disease progression within 12 months of neoadjuvant or adjuvant treatment with platinum-containing chemotherapy [94]. The indication reported is the same as the other PD-L1/PD-1 inhibitors. The approval was based on an updated data of a phase I/II multicenter, open-label study, Study-1108 of 182 patients with locally advanced or metastatic urothelial carcinoma whose disease progressed after prior platinum-containing chemotherapy. Durvalumab was administered at 10 mg/kg intravenously every 2 weeks for up to 12 months, or until unacceptable toxicity or disease progression. Confirmed ORR as assessed by blinded independent central review per RECIST 1.1, was 17.0% (95% CI 11.9, 23.3). At the data cutoff for the ORR analysis, median response duration was not reached (range 0.9 + to 19.9 + months). ORR was also analyzed by PD-L1 expression status as measured by VENTANA PD-L1 (SP263) Assay. In the 182 patients, the confirmed ORR was 26.3% (95% CI 17.8, 36.4) in 95 patients with a high PD-L1 score and 4.1% (95% CI 0.9, 11.5) in 73 patients with a low or negative PD-L1 score. This finding did not limit the indication of durvalumab to patients with high PD-L1 score.

#### Avelumab

To date, avelumab, an anti-PD-L1 antibody, is the latest addition to the armamentarium for the treatment of platinum-refractory urothelial carcinoma. In a single-arm, open-label JAVELIN Solid Tumor trial, avelumab showed the overall response rate of 13.3% (95% CI 9.1–18.4) among 226 patients who had been followed for at least 13 weeks. Patients received avelumab, 10 mg/kg intravenously, every 2 weeks until radiographic or clinical progression or unacceptable toxicity. All patients received pre-medication with an anti-histamine and acetaminophen prior to each avelumab administration. Confirmed overall response rate in patients who had been followed for at least 13 weeks was 13.3% (n = 30) (95% CI 9.1, 18.4), and 16.1% (n = 26) (95% CI 10.8, 22.8) in patients who had been followed for at least 6 months. Median time to response was 2.0 months (range 1.3–11.0). The median response duration had not been reached in patients followed for at least 13 weeks or at least 6 months, respectively, but ranged from 1.4 + to 17.4 + months in the two groups. Deaths due to an adverse reaction occurred in 6% of patients, who experienced either pneumonitis, respiratory failure, sepsis/urosepsis, cerebrovascular accident, or gastrointestinal adverse events. Serious adverse reactions were reported in 41% of patients. The most frequent serious adverse reactions reported in 2% or more of patients were urinary tract infection/urosepsis, abdominal pain, musculoskeletal pain, creatinine increased/renal failure, dehydration, hematuria/urinary tract hemorrhage, intestinal obstruction/small intestinal obstruction, and pyrexia. The recommended dose of avelumab is 10 mg/kg as an intravenous infusion over 60 min every 2 weeks. Unlike the other immune checkpoint inhibitors, avelumab requires premedication with an anti-histamine and acetaminophen prior to the first four infusions.

#### Pembrolizumab

To date, pembrolizumab remains investigational for urothelial carcinoma. The FDA approval is currently pending. Nevertheless, pembrolizumab is the first PD-1 inhibitor that has demonstrated a survival benefit over a standard chemotherapy in patients with platinum-refractory advanced urothelial carcinoma [95]. The study, KEYNOTE-045 was a randomized phase III study comparing pembrolizumab at a dose of 200 mg IV every 2 weeks with the investigator’s choice chemotherapy with paclitaxel, docetaxel, or vinflunine. The coprimary end points were overall survival and progression-free survival. The study showed statistically significant difference in the median overall survival in the total population 10.3 months (95% confidence interval [CI], 8.0–11.8) in the pembrolizumab group, as compared with 7.4 months (95% CI 6.1–8.3) in the chemotherapy group. The hazard ratio for death was 0.73; 95% CI 0.59–0.91; p = 0.002). Interestingly, the median OS among patients who had a tumor PD-L1 combined positive score of 10% or more was 8.0 months (95% CI 5.0–12.3) in the pembrolizumab group, as compared with 5.2 months (95% CI 4.0–7.4) in the chemotherapy group (hazard ratio, 0.57; 95% CI 0.37–0.88; p = 0.005). No significant difference was seen in the second co-primary endpoint, progression-free survival in the total population (HR: 0.98; 95% CI 0.81–1.19; p = 0.42). Fewer treatment-related adverse events of any grade were reported in the pembrolizumab group than in the chemotherapy group (60.9% vs. 90.2%); there were also fewer events of grade 3, 4, or 5 severity reported in the pembrolizumab group than in the chemotherapy group (15.0% vs. 49.4%). The study data is currently under review for the FDA approval.

The clinical activity of pembrolizumab in patients with metastatic urothelial carcinoma was first evaluated on a phase Ib study, KEYNOTE-012. In that study, patients were required to have at least 1% PD-L1 expression detected on the tumour cells or in tumour stroma, as determined by immunohistochemistry. Patients were given 10 mg/kg intravenous pembrolizumab every 2 weeks until disease progression, unacceptable toxic effects, or the end of the study (i.e., 24 months of treatment). Among 27 response evaluable patients, after a median follow-up of 13 months, an overall response was achieved in seven (26% [95% CI 11–46]) of 27 assessable patients, with three (11% [2–29]) complete and four (15% [4–34]) partial responses. None of the four deaths occurring during the study (cardiac arrest, pneumonia, sepsis, and subarachnoid hemorrhage) were deemed treatment related.

### Summary

UC is an immunotherapy-responsive disease. Since the first approval of atezolizumab in May 2016, three other PD-L1/PD-L1 inhibitors (nivolumab, durvalumab, and avelumab) have received accelerated approvals for treatment of platinum-refractory metastatic urothelial carcinoma, based on the data from their respective phase I/II studies demonstrating an ORR ranging from 13 to 19%. Pembrolizumab, pending approval by the FDA, has shown statistically significant survival difference of 2.9 months (10.3 months in the pembrolizumab group, as compared with 7.4 months. Additionally, atezolizumab has also recently received another clinical indication as a frontline therapy for patients who are unfit to receive cisplatin-based first line chemotherapy, based on a phase II study data demonstrating ORR of 24%. Despite these advances, objective responses are seen only in a fraction of our patients. Additionally, no biomarker tests are available to choose appropriate patient population and to choose PD-L1/PD-1 inhibitor to use. PD-L1 expression status does not reliably predict response or resistance to a PD-L1/PD-1 inhibitor. Future studies are needed to expand the clinical activity of these agents to a broader population by discovering new therapeutic targets and by combining with other immune therapy or conventional therapy.

## Role of immunotherapy in breast cancer

Breast cancer is the most common malignancy in women worldwide, and over 1.7 million cases are diagnosed each year globally [96]. Approximately 15% of breast cancers are triple-negative, defined as tumors which lack expression of ER, PR, and HER2 [97] and thus do not benefit from available targeted therapies. TNBC is more frequently seen in those of African and Hispanic ancestry, and is associated with an earlier age at diagnosis, an advanced stage at diagnosis, and a worse clinical outcome as compared to non-TNBCs. TNBC continues to represent an important clinical challenge, and new treatment strategies are urgently needed.

A number of observations over the past several years have led to the initial investigations of immunotherapy for the treatment of TNBC. Using TCGA data, Mittendorf et al. demonstrated that TNBCs have higher levels of PD-L1 mRNA expression (n = 120) as compared to non-TNBCs (n = 716), with 19% of TNBC tumors (n = 105) expressing PD-L1 by IHC [98].

Gene expression profiling has identified 6 TNBC subtypes, including an immunomodulatory, in addition to 2 basal-like (BL1 and BL2), a mesenchymal, a mesenchymal stem-like, and a luminal androgen receptor subtype [99]. The immunomodulatory subtype, which accounts for some 20% of TNBCs, is characterized by the elevated expression of genes involved in T cell function. Compared to other forms of breast cancer, TNBC has the most robust tumor immune infiltrate, suggesting that a subset of TNBCs are immunogenic [100].

PD-L1 expression has also been noted in hormone receptor positive breast cancer (HR+). HR+ breast cancer accounts for over 50% of all breast cancers, and 4–20% of HR+ breast cancers express PD-L1.

A handful of clinical trials of immune checkpoint inhibitors in advanced breast cancer have been reported to date, including trials of both PD-1 and PD-L1 inhibitors as monotherapy or in combination with chemotherapy. KEYNOTE-012 was a multicenter, phase Ib trial of pembrolizumab in patients with advanced PD-L1-positive (expression in stroma or ≥ 1% of tumor cells by immunohistochemistry) TNBC, gastric cancer, UC, and head and neck cancer. Among the 111 patients with TNBC prescreened for this study, 58.6% had PD-L1-positive disease. Thirty-two women (median age, 50.5 years; range 29–72 years) were enrolled and assessed for safety and antitumor activity. Common toxicities were mild, and similar to those observed in other tumor cohorts (e.g., arthralgia, fatigue, myalgia, and nausea); 15.6% patients reported grade ≥ 3 toxicity and one on study death, likely related to rapid disease progression. Among the 27 patients who were evaluable for antitumor activity, the overall response rate was 18.5%, the median time to response was 17.9 weeks (range 7.3–32.4 weeks), and the median duration of response was not yet reached (range 15.0–47.3 weeks) [9].

Preliminary results of the hormone-receptor positive (HR+) cohort of the phase Ib KEYNOTE-028 trial were presented at the 2015 San Antonio Breast Cancer Symposium. Of the 261 patients with HR+ advanced breast cancer prescreened for this study, 19.4% had PD-L1-positive disease. Among the 25 patients enrolled, 12% had a response to pembrolizumab monotherapy, 16% had stable disease, 60% had disease progression, and the remaining were unevaluable for response. The clinical benefit rate (response rate plus stable disease) was 20%; responses were durable in this group, with all 3 responders remaining on therapy in response for greater than 6 months [101].

Atezolizumab, an anti-PD-L1 antibody, has also been investigated as monotherapy in advanced breast cancer. It has been tested in metastatic TNBC as part of a multicenter Phase Ia study [102]. Tumors were determined to be PD-L1-positive if > 5% of tumor-infiltrating immune cells (IC) expressed PD-L1 (using the SP142 antibody). Fifty-four patients were enrolled in the TNBC cohort (median age 53 years, range 29–82 years), and 21 were evaluable for response at the time of initial presentation. Sixty-nine of those enrolled had PD-L1 positive tumors. Treatment-related AEs occurred in 63% of pts, most frequently fatigue (15%), pyrexia (15%), and nausea (15%). Eleven percent of patients experienced grade 3–5 related adverse events, and there were 2 study-related deaths. Among 21 efficacy-evaluable PD-L1-positive patients, the progression on their initial imaging evaluation, and subsequently went on to experience a durable shrinkage of both target and new lesions. Response duration ranged from 18 to 56 weeks, with the median not yet reached [102].

Adams and colleagues studied atezolizumab in combination with nab-paclitaxel in metastatic PD-L1-positive and PD-L1-negative TNBC [103]. In this phase 1b study, 32 patients received concurrent treatment with nab-paclitaxel and atezolizumab. The primary endpoint of the study was safety, with key secondary endpoints of ORR, duration of response, and progression-free survival. The median age of patients in the study was 55.5 years, and all patients had an ECOG PS 0-1. Patients could have received up to 3 prior systemic therapies for metastatic breast cancer, with 9 of the 24 evaluable patients receiving treatment in frontline metastatic setting. Eighty-seven percent of patients had previously received a taxane, for either early or advanced stage disease. At the time of the data cut-off, all 32 patients were evaluable for safety, and 24 patients were evaluable for efficacy. Grade 3/4 adverse events occurred in 56% of patients, with the most common including neutropenia (41%), thrombocytopenia (9%), and anemia (6%). Across all lines of therapy, the confirmed ORR was 41.7%, with a complete response rate of 4.2%. An additional 20.8% had stable disease, for an overall disease control rate of 62.5%. At the time of data cutoff, 11 of the 17 responses (65%) remained ongoing. In the second-line setting, the confirmed ORR was 25%, and in the third-line and beyond the ORR was 28.6%. In patients with PD-L1-positive TNBC, the ORR was 77.8% and the stable disease rate was 22.2%; in the PD-L1-negative group, the ORR was 57.1% and the stable disease rate was 42.9% [103].

The JAVELIN trial explored the efficacy and safety of the anti-PD-L1 antibody avelumab in patients with locally advanced or metastatic breast cancer [104]. This study enrolled 168 patients with MBC unselected for PD-L1 expression. Fifty-eight patients had TNBC, 72 had HR-positive/HER2-negative disease, 26 had HER2-positive disease, and 12 patients had unknown receptor status. Avelumab was given at 10 mg/kg intravenously every 2 weeks until disease progression or unacceptable toxicity. The overall response rate was low at 4.8% across the entire study population. Response rates in HR-positive, HER-2 positive and TNBC were 2.8, 3.8, and 8.6%, respectively. Response rates to avelumab were higher in those with PD-L1-positive tumours (defined as > 10% of immune cell hotspots), with 33% of those having PD-L1-positive disease experiencing a response. Furthermore, 5 of the 9 patients (44.4%) with PD-L1-positive TNBC had a response to therapy. The safety profile of avelumab was acceptable, with grade 3 or higher treatment-related adverse events only occurring in 13.7% of patients.

In conclusion, anti-PD-1/PD-L1 monotherapy is associated with a modest response rate in metastatic breast cancer. The addition of chemotherapy is associated with higher response rates. Response rates appear to be higher in those with PD-L1-positive tumors, in at least in some of the studies reported thus far. The trials, however, have used different antibodies and cutpoints for determining PD-L1 positivity, and a uniform method to define PD-L1 positivity in breast cancer is needed. Immune checkpoint inhibitors are safe and tolerable, and most of side effects are mild and easily managed. Future studies will investigate combination strategies, with the goal of building on the modest response rates observed with anti-PD-1/PD-L1 monotherapy. Trials studying immune checkpoint inhibitors in combination with other targeted agents, chemotherapy, and radiation therapy are ongoing. Many large randomized phase 2 and phase 3 registrational trials are ongoing, and results will be available in the near future.

## Abbreviations

ALK: anaplastic lymphoma kinase; APC: antigen presenting cell; CI: confidence interval; CNS: central nervous system; CT: computed tomography; CR: complete response; CTLA-4: Cytotoxic T-Lymphocyte Antigen4; EGFR: epidermal growth factor receptor; EOC: epithelial ovarian cancer; GC: gemcitabine/cisplatin; GITR: glucocorticoid-induced tumor necrosis factor receptor; G-CSF: granulocyte colony-stimulating factor; HR: hazard ratio; HNSCC: head and neck squamous cell carcinoma; HCC: hepatocarcinoma; HVEM: herpesvirus entry mediator; HR+: hormone-receptor positive; HER: human epidermal growth factor receptor; irAEs: immune-related adverse events; IHC: immunohistochemistry; LAG-3: lymphocyte antigen gene 3; mTOR: mammalian target of rapamycin; MVAC: methotrexate, vinblastine, adriamycin and cisplatin; MSI: microsatellite instability; NK: natural killer; NSCLC: non-small-cell lung carcinoma; ORR: objective response rate; OS: overall survival; PD-1: programmed death-1; PD-L1: programmed death ligand-1; PFS: progression free survival; Tregs: regulatory T cells; TNBC: triple-negative breast cancer; TIL: tumor-infiltrating lymphocytes; TKI: tyrosine kinase inhibitor; UC: urothelial carcinoma.

### Immunotherapeutic agents

Atezolizumab: anti-PD-L1 IgG1 humanized monoclonal Ab; Avelumab: anti-PD-L1 IgG1 human monoclonal Ab; Durvalumab: anti-PD-L1 IgG1 κ human monoclonal Ab; Ipilimumab: anti-CTLA-4 IgG1 human monoclonal Ab; Nivolumab: anti-PD1 IgG4 human monoclonal Ab; Pembrolizumab: anti-PD-1 IgG4 κ humanized monoclonal Ab.
